# Lysine 164 is critical for SARS-CoV-2 Nsp1 inhibition of host gene expression

**DOI:** 10.1099/jgv.0.001513

**Published:** 2020-11-05

**Authors:** Zhou Shen, Guangxu Zhang, Yilin Yang, Mengxia Li, Siqi Yang, Guiqing Peng

**Affiliations:** ^1^​ State Key Laboratory of Agricultural Microbiology, College of Veterinary Medicine, Huazhong Agricultural University, Wuhan, PR China; ^2^​ Key Laboratory of Preventive Veterinary Medicine in Hubei Province, The Cooperative Innovation Center for Sustainable Pig Production, PR China

**Keywords:** 40S ribosomal subunit, Host gene expression inhibition, K164, Nonstructural protein 1, Severe acute respiratory syndrome coronavirus 2

## Abstract

The emerging pathogen severe acute respiratory syndrome coronavirus 2 (SARS-CoV-2) has caused social and economic disruption worldwide, infecting over 9.0 million people and killing over 469 000 by 24 June 2020. Unfortunately, no vaccine or antiviral drug that completely eliminates the transmissible disease coronavirus disease 2019 (COVID-19) has been developed to date. Given that coronavirus nonstructural protein 1 (nsp1) is a good target for attenuated vaccines, it is of great significance to explore the detailed characteristics of SARS-CoV-2 nsp1. Here, we first confirmed that SARS-CoV-2 nsp1 had a conserved function similar to that of SARS-CoV nsp1 in inhibiting host-protein synthesis and showed greater inhibition efficiency, as revealed by ribopuromycylation and Renilla luciferase (Rluc) reporter assays. Specifically, bioinformatics and biochemical experiments showed that by interacting with 40S ribosomal subunit, the lysine located at amino acid 164 (K164) was the key residue that enabled SARS-CoV-2 nsp1 to suppress host gene expression. Furthermore, as an inhibitor of host-protein expression, SARS-CoV-2 nsp1 contributed to cell-cycle arrest in G0/G1 phase, which might provide a favourable environment for virus production. Taken together, this research uncovered the detailed mechanism by which SARS-CoV-2 nsp1 K164 inhibited host gene expression, laying the foundation for the development of attenuated vaccines based on nsp1 modification.

## Introduction

Coronaviruses (CoVs) are classified into four genera, alpha-CoV, beta-CoV, gamma-CoV and delta-CoV, which cause a variety of diseases [1, 2]. To date, seven types of CoVs that can infect humans have been identified, and three highly pathogenic diseases are attributed to beta-CoVs [3, 4]. Severe acute respiratory syndrome coronavirus (SARS-CoV), a lineage B beta-CoV, has infected more than 8000 individuals and resulted in approximately 800 fatalities [5, 6]. Middle East respiratory syndrome coronavirus (MERS-CoV) is a lineage C beta-CoV from dromedary camels, currently with 2494 confirmed cases and 858 deaths [7, 8]. In contrast, severe acute respiratory syndrome coronavirus 2 (SARS-CoV-2), a lineage B beta-CoV, has caused a worldwide pandemic for the past several months, with unprecedented challenges to the economy and public health [9, 10]. Furthermore, the difficulties in screening asymptomatic infection cause this pandemic to be more difficult to prevent and control [11, 12]. According to World Health Organization (WHO) statistics, more than 9.0 million people have been infected with SARS-CoV-2 as of 24 June 2020, with 469 000 deaths (https://www.who.int/emergencies/diseases/novel-coronavirus-2019). Moreover, those in resource-poor regions remain undertested, and disease occurrence is underreported [13]. This urgent situation requires the immediate development of effective prophylactics or therapeutics to prevent SARS-CoV-2 infection. Due to the rapid spread of SARS-CoV-2 and the lack of specific treatment, many efforts have focused on the development of neutralizing antibodies and vaccines [14–16]. The glycoprotein spike (S), a main target for neutralizing antibodies, contains a receptor-binding domain (RBD) that mediates membrane fusion and virus entry [14]. Similar to SARS-CoV, SARS-CoV-2 recognizes angiotensin-converting enzyme 2 (ACE2) as its host receptor, and many previous antibodies or peptide inhibitors specific to SARS-CoV have been shown to be effective against SARS-CoV-2 S protein-mediated membrane fusion [17–19]. However, due to the incomplete understanding of the pathogenic mechanism of SARS-CoV-2, it is difficult to quickly and effectively control its spread.

CoV nsp1, the N-terminal protein encoded by gene 1, is an important virulence factor in both alpha-CoVs and beta-CoVs. With regard to alpha-CoVs, we confirmed that modifying the critical motif (91–95 aa) of transmissible gastroenteritis virus (TGEV) nsp1 did not affect replication capacity in cell culture but significantly reduced pathogenicity in pigs [20]. Among beta-CoVs, deleting the critical region of SARS-CoV nsp1 and the region encoding amino acids 277 to 309 from the murine hepatitis virus (MHV) nsp1 gene were useful for developing attenuated vaccines for beta-CoVs [21–23]. Although the entire genome of SARS-CoV-2 is approximately 80 % amino acid sequence identical to that of SARS-CoV [10], it remains unclear whether SARS-CoV-2 nsp1 possesses biological functions or mechanisms similar to those of SARS-CoV nsp1. In this study, we show that compared to SARS-CoV nsp1, SARS-CoV-2 nsp1 exhibits a much higher capacity to inhibit the synthesis of host proteins, which may contribute to the enhanced pathogenicity of SARS-CoV-2. Based on Rluc and ribopuromycylation assays, H165 of SARS-CoV-2 nsp1 partially contributes to the inhibitory activity of nsp1, but K164 is the key residue related to host gene expression inhibition. Using co-immunoprecipitation (Co-IP) experiments, we show that SARS-CoV-2 nsp1 is likely to regulate host-protein synthesis by binding to 40S ribosomal subunits, similar to SARS-CoV nsp1 [24]. Furthermore, mutations at K164 result in loss of interaction with 40S ribosomal subunits, further indicating that K164 is involved in inhibiting host-protein synthesis. Although SARS-CoV-2 nsp1 does not affect cell vitality, it arrests the cell cycle in G0/G1 phase, which may provide a favourable environment for virus production. Overall, our findings provide novel insight into the limited knowledge on the molecular mechanisms of SARS-CoV-2 nsp1.

## Results

### SARS-CoV-2 nsp1 shows an obvious capacity to inhibit the synthesis of host proteins

**Fig. 1.**
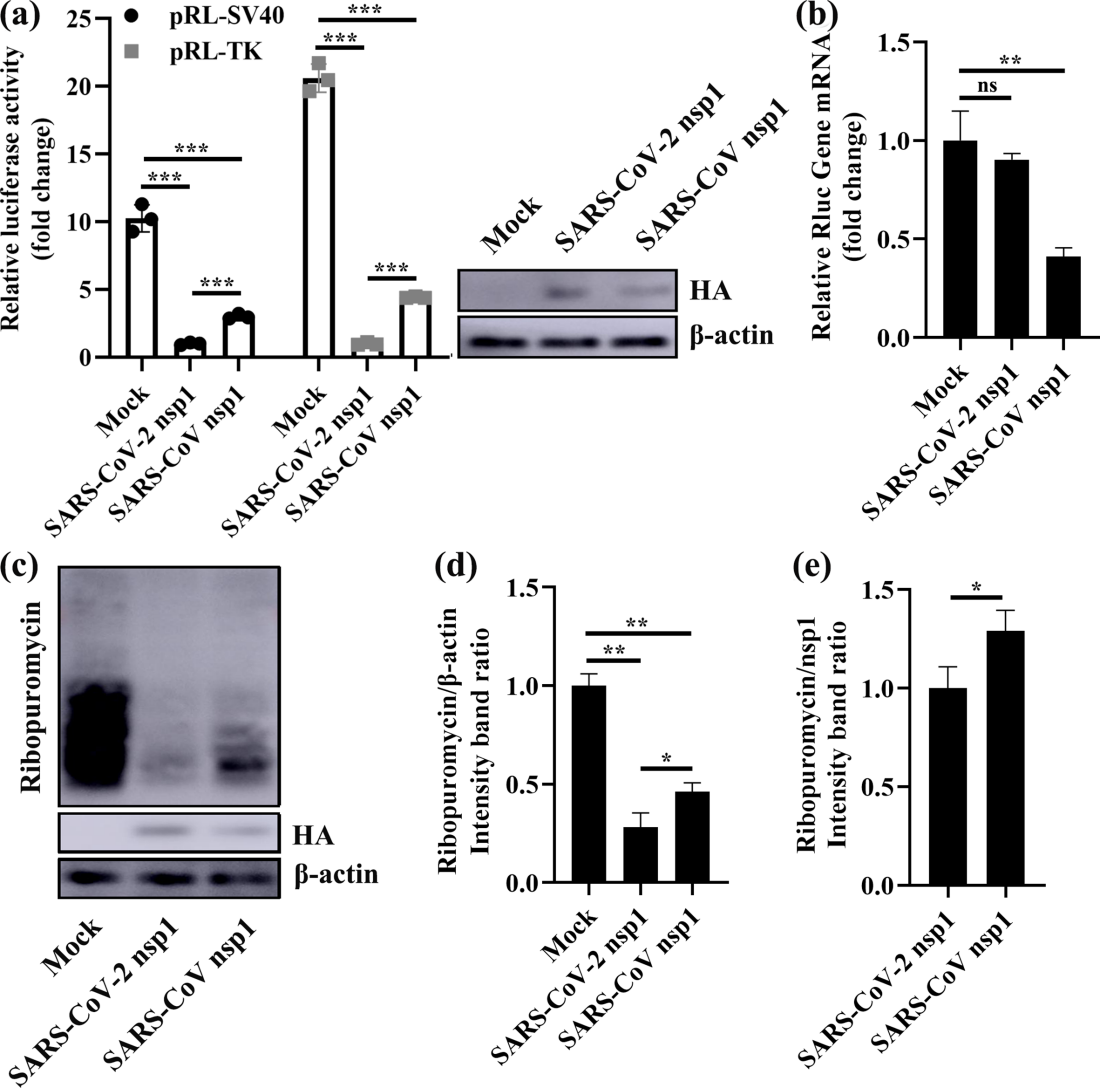
SARS-CoV-2 nsp1 inhibits host-protein synthesis. (a) Luciferase activities in HEK-293T cells transfected with 0.5 µg of either PCAGGS-SARS-CoV-2 nsp1 (SARS-CoV-2 nsp1), PCAGGS-SARS-CoV nsp1 (SARS-CoV nsp1) or PCAGGS (Mock) together with 0.2 µg of the indicated reporter plasmid (pRL-TK or pRL-SV40) were determined at 36 h post-transfection after standardization with cells expressing SARS-CoV-2 nsp1. Error bars show the standard deviation (SD) of the results from three independent experiments. Cell extracts were also subjected to Western-blot analysis using an anti-HA antibody (top) or anti-β-actin antibody (bottom). ***, *P* <0.001. (b) HEK-293T cells were cotransfected with pRL-SV40 encoding the Rluc reporter gene downstream of the SV40 promoter and one of the following plasmids: PCAGGS (mock), PCAGGS-SARS-CoV-2 nsp1 (SARS-CoV-2 nsp1) or PCAGGS-SARS-CoV nsp1 (SARS-CoV nsp1), respectively. At 24 h post-transfection, the cells were lysed and subjected to real-time quantitative PCR analysis. The values of SARS-CoV-2 nsp1 and SARS-CoV nsp1 were normalized to those of the untreated empty vector (PCAGGS) control, which was set to 1 (*n*=3). Asterisks indicate statistical significance calculated based on Student’s *t*-test. ns, not significant; **, *P* <0.01. (c) HEK-293T cells were transfected with 2.5 µg of either PCAGGS-SARS-CoV-2 nsp1 (SARS-CoV-2 nsp1), PCAGGS-SARS-CoV nsp1 (SARS-CoV nsp1) or PCAGGS (Mock) plasmid. At 36 h post-transfection, the cells were pulsed with 3 µm puromycin for 1 h at 37 °C and then subjected to Western-blot analysis using an anti-puromycin antibody (top), anti-HA antibody (middle), or anti-β-actin antibody (bottom). (d and e) The grey scale values of the proteins were analysed by ImageJ (*n*=3). *, *P* <0.05; **, *P* <0.01.

Because the amino acid sequences of SARS-CoV-2 and SARS-CoV nsp1 were highly conserved, we sought to determine whether SARS-CoV-2 nsp1 also had the function of inhibiting host-protein synthesis, similar to SARS-CoV nsp1 [23]. To this end, we cotransfected human embryonic kidney 293 (HEK-293T) cells with the plasmid pRL-SV40 or pRL-TK expressing the constitutive promoter-driven Renilla luciferase (Rluc) gene, together with one of the following plasmids: PCAGGS (Mock), PCAGGS-SARS-CoV-2 nsp1 (express SARS-CoV-2 nsp1 protein) or PCAGGS-SARS-CoV nsp1 (express SARS-CoV nsp1 protein). The SARS-CoV-2 and SARS-CoV nsp1 proteins contained an N-terminal HA epitope tag. The results showed that both SARS-CoV-2 and SARS-CoV nsp1 significantly inhibited expression of the Rluc gene (Fig. 1a). Specifically, coexpression of SARS-CoV-2 nsp1 or SARS-CoV nsp1 reduced induction of the SV40 promoter by a factor of ∼10 or ∼2.5 times, respectively. Under induction of the TK promoter, coexpression of SARS-CoV-2 nsp1 or SARS-CoV nsp1 was reduced by a factor of ∼20 and ∼5 times, respectively. Interestingly, the ability of SARS-CoV-2 nsp1 to inhibit Rluc gene expression was ∼fourfold higher than that of SARS-CoV nsp1. We also detected nsp1 in HEK-293T cells by Western-blot assays and found that expression of both SARS-CoV-2 and SARS-CoV nsp1 was very low (Fig. 1a). This low-level accumulation suggested that the nsp1 proteins of SARS-CoV-2 and SARS-CoV inhibited their own expression. Then we investigated whether SARS-CoV-2 nsp1 induced degradation of host mRNAs by real-time quantitative PCR. Using SARS-CoV nsp1 as a positive control, we found that the SARS-CoV-2 nsp1 had no effect on the degradation of Rluc mRNA (Fig. 1b). By labelling newly produced proteins of nsp1-transfected cells and mock-transfected cells with puromycin, we found that SARS-CoV-2 nsp1 and SARS-CoV nsp1 broadly inhibited host-protein synthesis (Fig. 1c). Furthermore, the inhibitory effect of SARS-CoV-2 nsp1 was stronger than that of SARS-CoV nsp1 (Fig. 1d, e), which was consistent with the Rluc assay results. Thus, we concluded that SARS-CoV-2 nsp1 had a stronger effect on inhibiting host-protein synthesis than SARS-CoV nsp1.

### K164 and H165 are conserved important amino acids of SARS-CoV-2 and SARS-CoV nsp1

**Fig. 2.**
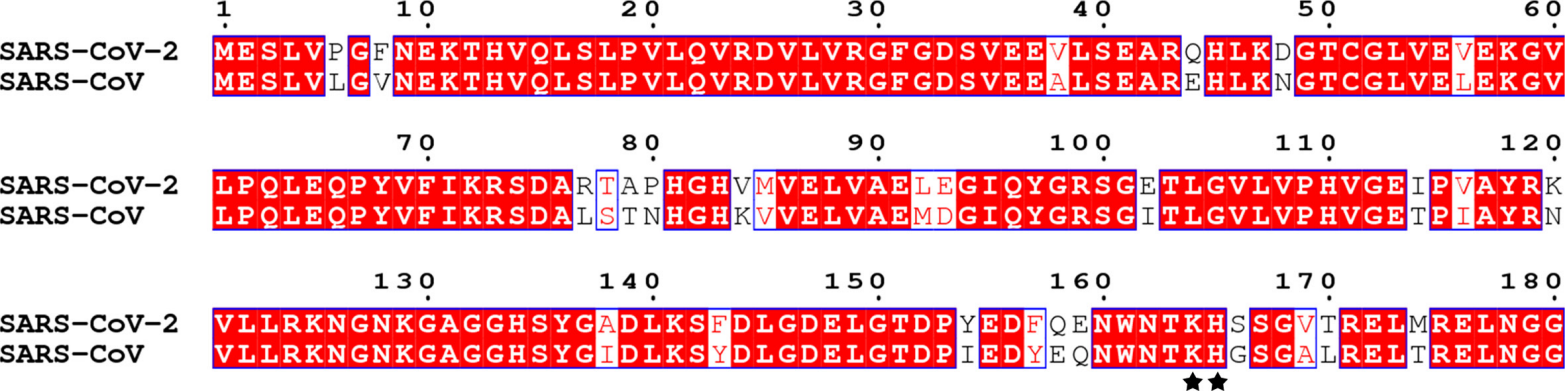
Multiple sequence alignment of SARS-CoV-2 nsp1 and SARS-CoV nsp1. The following sequences from GenBank were for sequence alignment (given as the name, abbreviation and GenBank accession number): severe acute respiratory syndrome coronavirus 2 (SARS-CoV-2), NC_045512.2; severe acute respiratory syndrome coronavirus (SARS-CoV), NC_004718.3. The residue numbers with reference to SARS-CoV-2 nsp1 are labelled on top of the panel. K164 and H165 are marked by small black dots. Residues conserved in most sequences are shown in red and are boxed with a white background. The sequences were aligned with ClustalW2, and the figure was prepared with ESPript3.0.

**Fig. 3.**
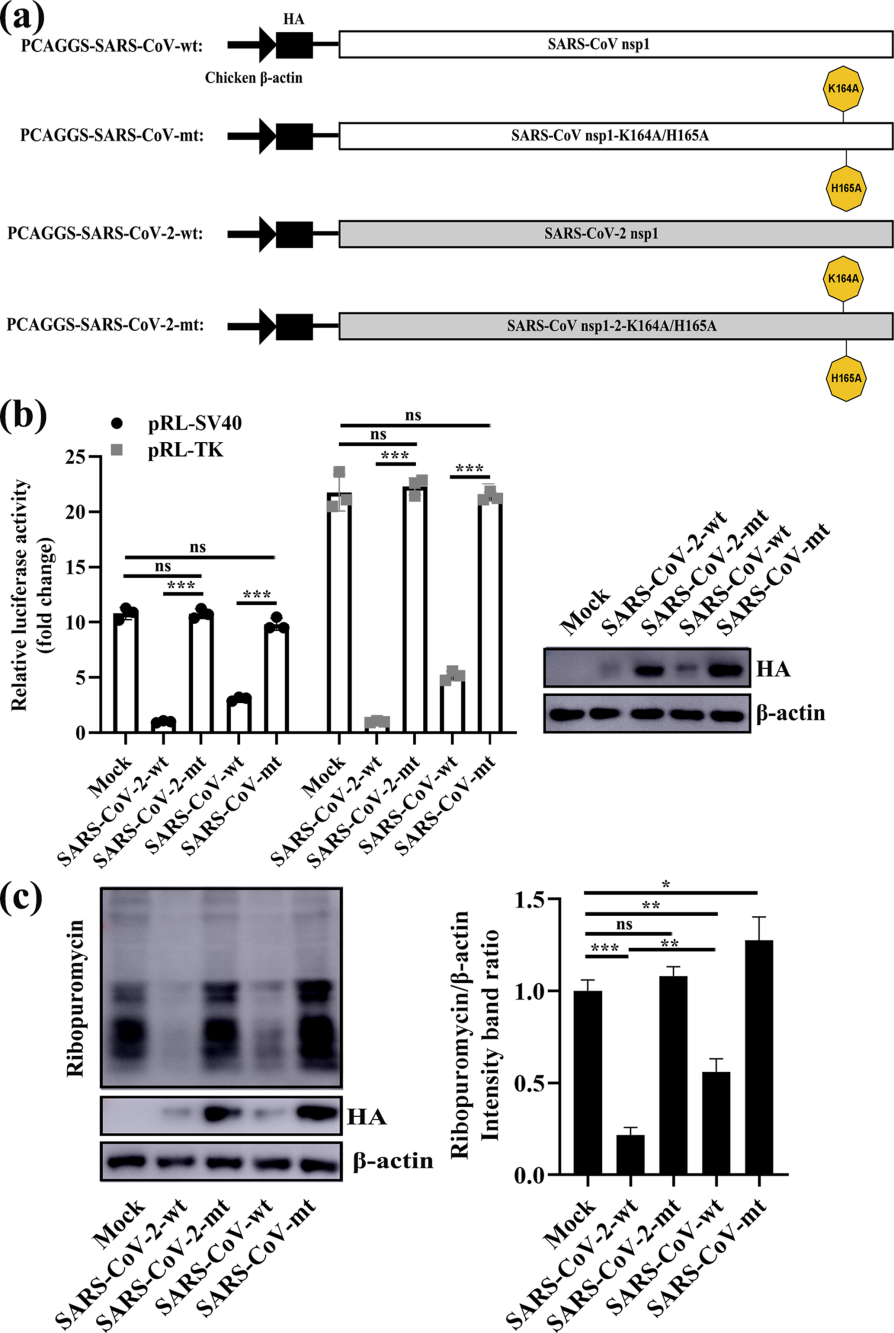
K164 and H165 are conserved important amino acids in SARS-CoV-2 and SARS-CoV nsp1. (a) Schematic diagrams of PCAGGS-SARS-CoV-wt (SARS-CoV nsp1), PCAGGS-SARS-CoV-mt (SARS-CoV nsp1-K164A/H165A), PCAGGS-SARS-CoV-2-wt (SARS-CoV-2 nsp1) and PCAGGS-SARS-CoV-2-mt (SARS-CoV-2 nsp1-K164A/H165A). (b) Luciferase activities in HEK-293T cells transfected with 0.5 µg of either PCAGGS-SARS-CoV-wt (SARS-CoV nsp1), PCAGGS-SARS-CoV-mt (SARS-CoV nsp1-K164A/H165A), PCAGGS-SARS-CoV-2-wt (SARS-CoV-2 nsp1), PCAGGS-SARS-CoV-2-mt (SARS-CoV-2 nsp1-K164A/H165A) or PCAGGS (mock) together with 0.2 µg of the indicated reporter plasmid (pRL-TK or pRL-SV40) were determined at 36 h post-transfection after standardization with cells expressing SARS-CoV-2 nsp1. Error bars show the SD of the results from three independent experiments. Cell extracts were also subjected to Western-blot analysis using an anti-HA antibody (top) or anti-β-actin antibody (bottom). ns, not significant; ***, *P* <0.001 (considered extremely significant). (c) HEK-293T cells were transfected with 2.5 µg of either PCAGGS-SARS-CoV-wt (SARS-CoV nsp1), PCAGGS-SARS-CoV-mt (SARS-CoV nsp1-K164A/H165A), PCAGGS-SARS-CoV-2-wt (SARS-CoV-2 nsp1), PCAGGS-SARS-CoV-2-mt (SARS-CoV-2 nsp1-K164A/H165A) or PCAGGS (mock) plasmids. At 36 h post-transfection, the cells were pulsed with 3 µm puromycin for 1 h at 37 °C and then subjected to Western-blot analysis using an anti-puromycin antibody (top), anti-HA antibody (middle) or anti-β-actin antibody (bottom). The grey scale values of the proteins were analysed by ImageJ (*n*=3). ns, not significant; *, *P* <0.05; **, *P* <0.01; ***, *P* <0.001.

Because previous data indicated that mutation of the positively charged amino acids K164 and H165 of SARS-CoV nsp1 might abolish the biological functions of nsp1 [25], we next aligned the sequences of SARS-CoV-2 and SARS-CoV nsp1 and found that K164 and H165 are conserved amino acids in both nsp1s (Fig. 2). To determine the effect of K164 and H165 in SARS-CoV-2 nsp1 on host gene expression suppression, we first constructed PCAGGS-nsp1-mt, in which K164 and H165 in the C-terminal region were replaced with alanines and an HA tag was added to the N-terminus (Fig. 3a). HEK-293T cells were cotransfected with the above plasmids and a reporter plasmid, pRL-SV40 or pRL-TK, in which the Rluc gene was cloned downstream of the SV40 promoter or TK promoter. As controls, the parental plasmid, PCAGGS and PCAGGS-nsp1-WT encoding the full-length nsp1 protein (nsp1-WT) were used in place of PCAGGS-nsp1-mt (nsp1-K164A/H165A). Consistent with previous data [25], SARS-CoV nsp1-K164A/H165A did not suppress Rluc gene expression. Similar to SARS-CoV nsp1-K164A/H165A, the SARS-CoV-2 nsp1-K164A/H165A protein did not affect expression of Rluc activity. Western-blot analysis using an anti-HA antibody revealed efficient accumulation of nsp1-K164A/H165A compared to low levels of accumulation found with the nsp1-WT protein, which suggested that SARS-CoV-2 nsp1-K164A/H165A did not suppress expression of its own gene (Fig. 3b). Next, the effect of Nsp1-mt protein expression on host-protein synthesis was examined. HEK-293T cells were independently transfected with the PCAGGS, nsp1-WT and nsp1-MT plasmids; at 23 h after transfection, the cells were treated with puromycin for 1 h, and cell extracts were prepared and analysed by Western blotting. Consistent with a previous report [25], expression of the SARS-CoV nsp1-WT protein, but not the SARS-CoV-2 nsp1-K164A/H165A protein, strongly suppressed host-protein synthesis (Fig. 3c). In contrast, SARS-CoV-2 nsp1-K164A/H165A had little effect on host-protein synthesis, similar to that of SARS-CoV nsp1 (Fig. 3c). These data demonstrate that the amino acids K164 and H165 are important for the ability of SARS-CoV-2 to inhibit host gene expression.

### K164 is the key residue for SARS-CoV-2 nsp1 inhibition of host-protein synthesis

**Fig. 4.**
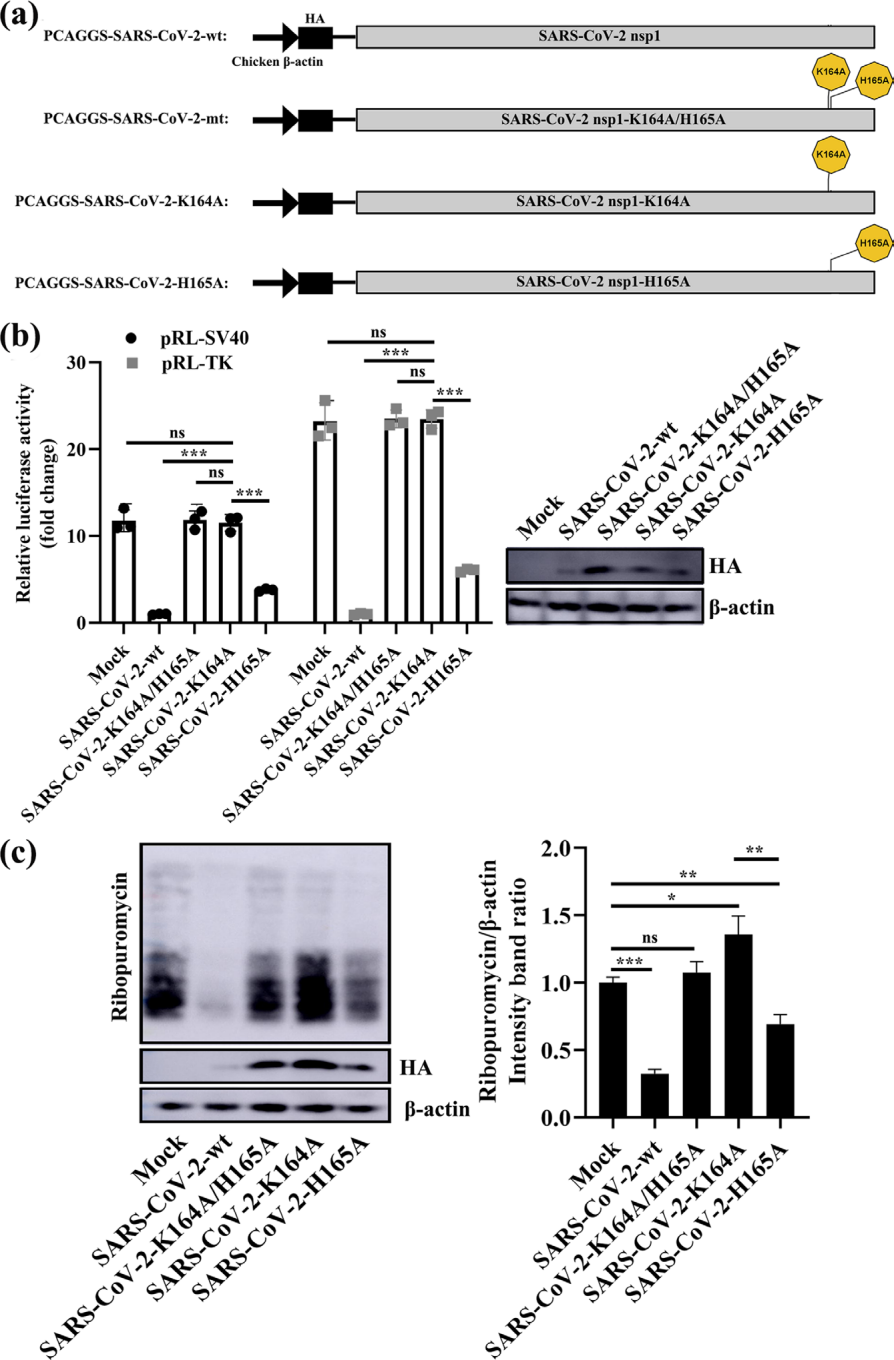
K164 is a key residue for the ability of SARS-CoV-2 nsp1 to inhibit host gene expression. (a) Schematic diagrams of PCAGGS-SARS-CoV-2-wt (SARS-CoV-2 nsp1), PCAGGS-SARS-CoV-2-mt (SARS-CoV-2 nsp1-K164A/H165A), PCAGGS-SARS-CoV-2-K164A (SARS-CoV-2 nsp1-K164A) and PCAGGS-SARS-CoV-2-H165A (SARS-CoV-2 nsp1-H165A). (b) Luciferase activities in HEK-293T cells transfected with 0.5 µg of either PCAGGS-SARS-CoV-2-wt (SARS-CoV-2 nsp1), PCAGGS-SARS-CoV-2-mt (SARS-CoV-2 nsp1-K164A/H165A), PCAGGS-SARS-CoV-2-K164A (SARS-CoV-2 nsp1-K164A), PCAGGS-SARS-CoV-2-H165A (SARS-CoV-2 nsp1-H165A) or PCAGGS (mock) together with 0.2 µg of the indicated reporter plasmid (pRL-TK or pRL-SV40) were determined at 36 h post-transfection after standardization with cells expressing SARS-CoV-2 nsp1. Error bars show the SD of the results from three independent experiments. Cell extracts were also subjected to Western-blot analysis using an anti-HA antibody (top) or anti-β-actin antibody (bottom). ns, not significant; ***, *P* <0.001 (considered extremely significant). (c) HEK-293T cells were transfected with 2.5 µg of either PCAGGS-SARS-CoV-2-wt (SARS-CoV-2 nsp1), p-SARS-CoV-2-mt (SARS-CoV-2 nsp1-K164A/H165A), PCAGGS-SARS-CoV-2-K164A (SARS-CoV-2 nsp1-K164A), PCAGGS-SARS-CoV-2-H165A (SARS-CoV-2 nsp1-H165A) or PCAGGS (mock) plasmids. At 36 h post-transfection, the cells were pulsed with 3 µm puromycin for 1 h at 37 °C and then subjected to Western-blot analysis using an anti-puromycin antibody (top), anti-HA antibody (middle) or anti-β-actin antibody (bottom). The grey scale values of the proteins were analysed by ImageJ (*n*=3). ns, not significant; *, *P* <0.05; **, *P* <0.01; ***, *P* <0.001.

To further examine the role of K164 and H165 in SARS-CoV-2 nsp1-mediated gene suppression, we constructed the plasmids PCAGGS-SARS-CoV-2-K164A and PCAGGS-SARS-CoV-2-H165A, carrying substitutions of K164 and H165 in nsp1, respectively, with alanine (Fig. 4a). HEK-293T cells transfected with either PCAGGS, PCAGGS-SARS-CoV-2-K164A/H165A, PCAGGS-SARS-CoV-2-K164A or PCAGGS-SARS-CoV-2-H165A together with pRL-SV40 or pRL-TK were assessed for Rluc activity at 24 h post-transfection. Although SARS-CoV-2-K164A/H165A and SARS-CoV-2-K164A failed to suppress expression of the Rluc gene, cells expressing SARS-CoV-2-K164A exhibited suppression that was substantial but weaker than that of SARS-CoV-2-wt (Fig. 4b). Furthermore, the ability of SARS-CoV-2-H165A to inhibit Rluc gene expression was ∼fourfold higher than that of SARS-CoV-2-K164A. To further study mutant proteins inhibiting host-protein synthesis, we used a ribopuromycylation experiment (Fig. 4c). The results showed that SARS-CoV-2-K164A completely lost the host-protein-synthesis inhibitory function, and SARS-CoV-2-H165A partially retained this function. These results suggest that K164 is responsible for suppressing protein synthesis.

### SARS-CoV-2 nsp1 inhibits host-protein synthesis through interaction of K164 with 40S ribosomal subunits

**Fig. 5.**
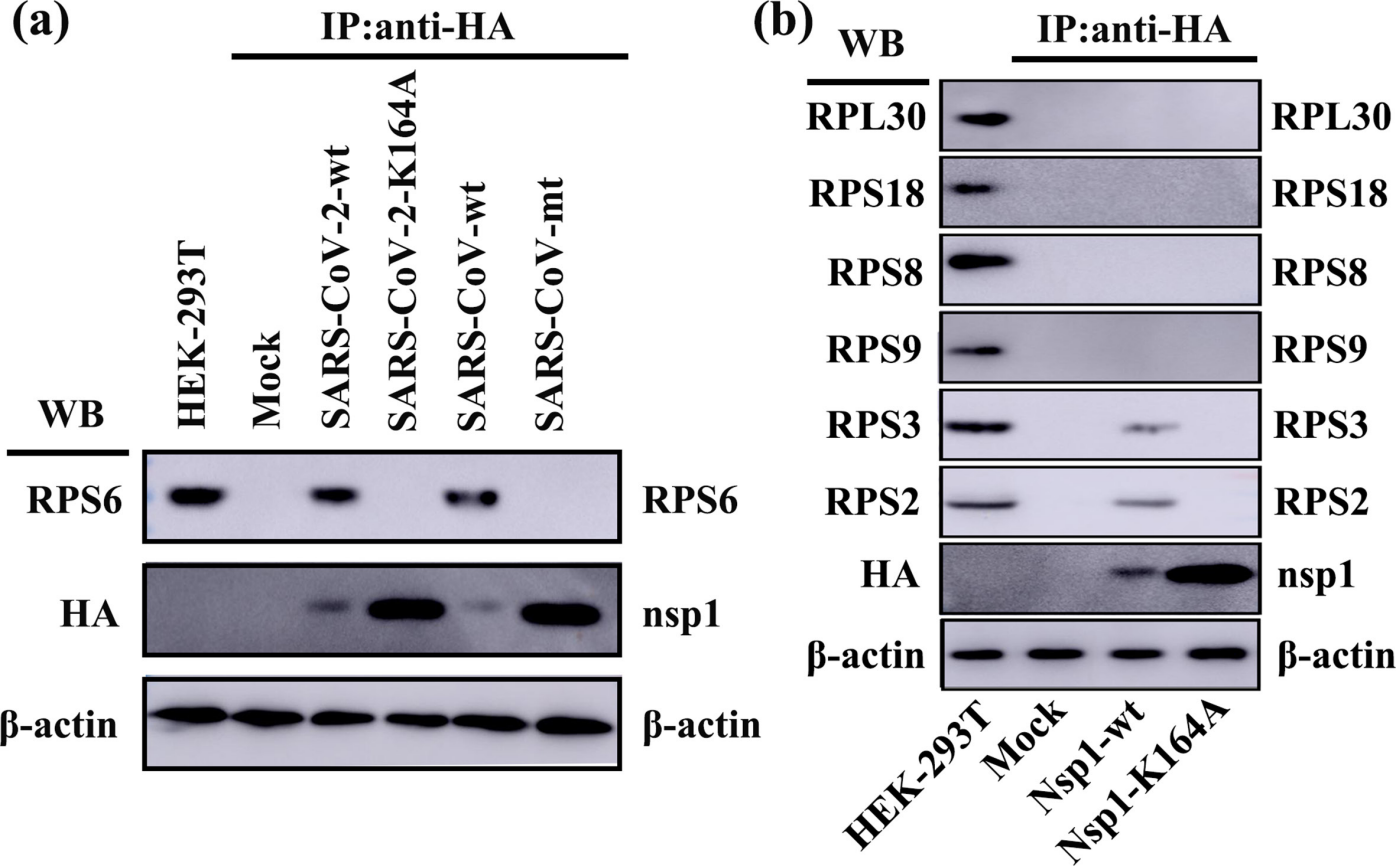
SARS-CoV-2 nsp1 K164A abolishes the interaction between nsp1 and the 40S ribosomal subunit in HEK-293T cells. (a) Using PCAGGS-SARS-CoV nsp1 (SARS-CoV-wt) and PCAGGS-SARS-CoV nsp1-K164A/H165A (SARS-CoV-mt) as a control, immunoprecipitated proteins were examined by Western-blot analysis using an anti-RPS6 antibody (40S subunit specific) and anti-HA antibody for PCAGGS (mock), PCAGGS-SARS-CoV-2 nsp1 (SARS-CoV-2-wt) and PCAGGS-SARS-CoV-2 nsp1-K164A (SARS-CoV-2-mt). (b) The immunoprecipitated proteins (RPS2, RPS3, RPS9, RPS8, RPS18 and RPL30) and SARS-CoV-2 nsp1 (Nsp1-wt) or SARS-CoV-2 nsp1-K164A (Nsp1-K164A) were examined by Western-blot analysis using anti-RPS2, anti-RPS3, anti-RPS9, anti-RPS8, anti-RPS18, anti-RPL30 and anti-HA antibody. The expression of β-actin was detected with an anti-β-actin monoclonal Ab to confirm equal protein loading.

We next explored the mechanism of host-protein synthesis regulation by K164 in SARS-CoV-2 nsp1. A previous study reported that SARS-CoV nsp1 inhibits host translation through interaction of K164 and H165 with 40S ribosomal subunits [24]. To examine whether K164 in SARS-CoV-2 nsp1 binds to RPS6 (a core component of the 40S ribosomal subunit) and inactivates its translational function, we used HEK-293T cells to carry out Co-IP experiments with HA beads and an anti-RPS6 antibody. Consistent with a previous report [24], efficient IP of RPS6 with nsp1, but not with PCAGGS and SARS-CoV nsp1-K164A/H165A (Fig. 5a), suggested the tight association of SARS-CoV nsp1 with RPS6 through K164 and H165. Moreover, we unexpectedly observed that SARS-CoV-2 nsp1 was able to pull down RPS6 in HEK-293T cells but that the K164 mutant protein could not, emphasizing that K164 was responsible for the inhibition of host-protein synthesis by SARS-CoV-2 nsp1. Meanwhile, we detected other small and large ribosomal subunits proteins (RPS2, RPS3, RPS8, RPS9, RPS18 and RPL30) using Co-IP experiments (Fig. 5b). The results showed that SARS-CoV-2 nsp1 interacted with RPS2 and RPS3, which was consistent with the current report [26]. Furthermore, the K164A could abolish binding to RPS2 and RPS3, which reflected that K164 played an important role in the interaction between nsp1 and 40S ribosomal subunits. Collectively, these data indicate that SARS-CoV-2 nsp1-mediated suppression of translation via inactivation of the translational functions of 40S ribosomal subunits is conserved with SARS-CoV nsp1.

### Expression of SARS-CoV-2 nsp1 induces G0/G1-phase arrest

**Fig. 6.**
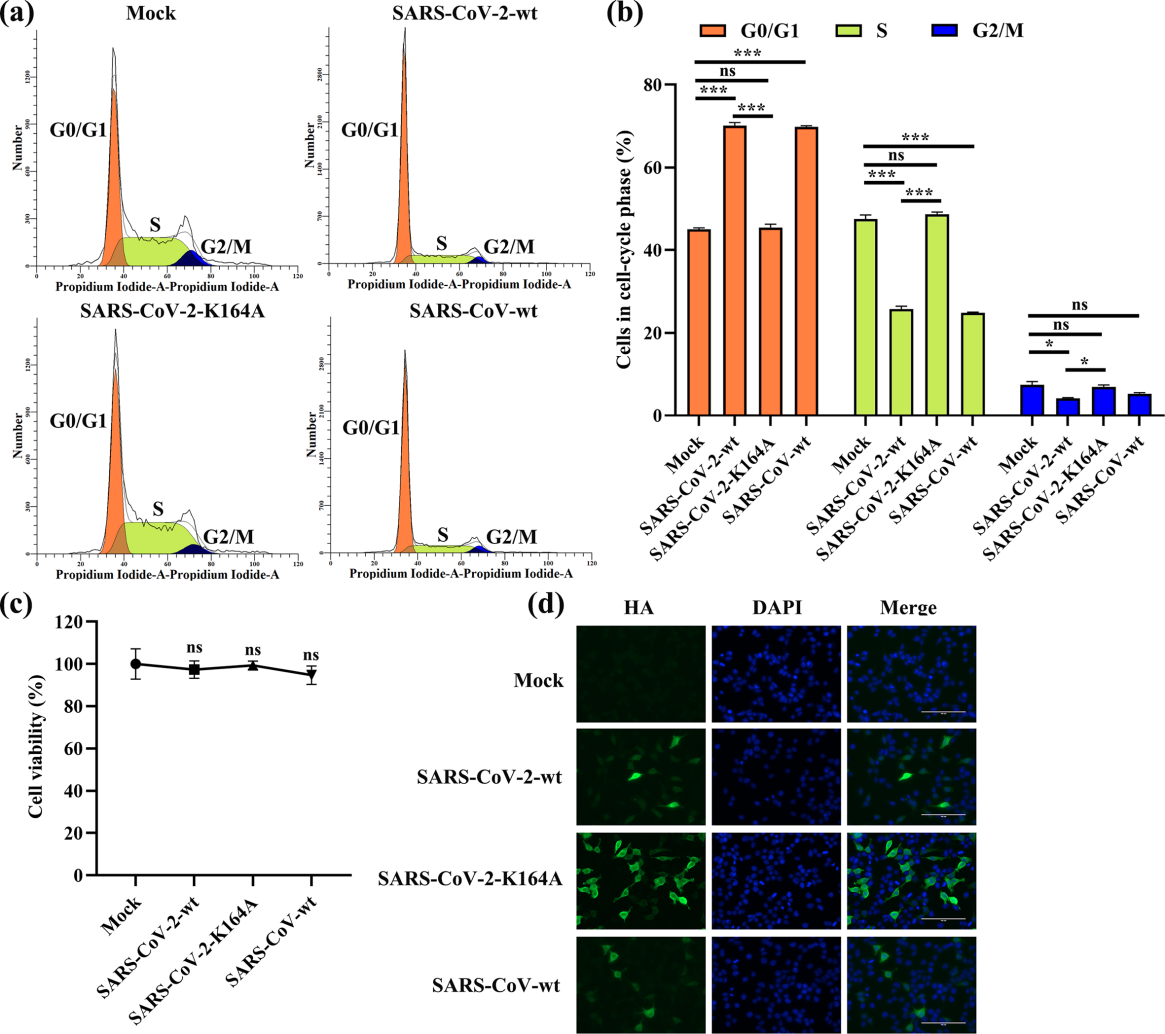
SARS-CoV-2 nsp1 promotes cell retention in G0/G1 phase. (a) HEK-293T cells transfected with PCAGGS (mock), p-SARS-CoV-2 nsp1-wt (SARS-CoV-2-wt), PCAGGS-SARS-CoV-2 nap1-K164A (SARS-CoV-2-K164A) or PCAGGS-SARS-CoV nsp1-wt (SARS-CoV-wt) for 36 h were fixed, stained with PI in the presence of RNase A, and analysed using flow cytometry to determine the DNA content and distribution of the cell population in various phases of the cell cycle. The data were analysed using ModFit software. (b) The data were analysed as described in (a). Error bars show the SD of the results from three independent experiments. The asterisks indicate the statistical significance calculated using Student’s *t*-test. ns, not significant; *, *P* <0.05; ***, *P* <0.001. (c) Cell-viability analysis of wild-type and mutant SARS-CoV-2 nsp1 in HEK-293T cells, as evaluated using a CellTiter-Glo luminescent cell-viability assay. Percent cell viability=100× (luminescence of the experimental group/luminescence of the control group). The data were analysed as described in (a). Error bars show the SD of the results from three independent experiments. (d) HEK-293T cells were transfected with PCAGGS (mock), PCAGGS-SARS-CoV-2 nsp1-wt (SARS-CoV-2-wt), PCAGGS-SARS-CoV-2 nap1-K164A (SARS-CoV-2-K164A) or PCAGGS-SARS-CoV nsp1-wt (SARS-CoV-wt) for 36 h. The cells were then fixed and subjected to an immunofluorescence assay with a mouse anti-HA antibody as the primary antibody, followed by staining with the secondary antibody Alexa Fluor 488-conjugated donkey anti-mouse IgG (green). DAPI staining (blue) indicates cell nuclei. Fluorescent images were acquired with an LSM 510 Meta confocal laser-scanning microscope (Carl Zeiss, Zena, Germany). Original magnification ×200 (scale bars 100 µm).

SARS-CoV nsp1 has been reported to promote host-cell retention in G0/G1 phase [22], suggesting that inhibition of host-protein expression contributes to cell-cycle arrest. To evaluate the effects of SARS-CoV-2 nsp1 and SARS-CoV-2 nsp1-K164A on cell proliferation, we transfected plasmids encoding individual viral nsp1 gene into HEK-293T cells. PCAGGS, SARS-CoV-2 nsp1, SARS-CoV-2 nsp1-K164A and SARS-CoV nsp1 were detected by anti-HA antibodies at 24 h post-transfection by indirect immunofluorescence assay (IFA) (Fig. 6d). Similar to SARS-CoV nsp1, expression of SARS-CoV-2 nsp1 increased the population of cells in G0/G1 phase by 26 % and decreased that in S phase by 24 % compared to the mock-transfected population (Fig. 6a, b). Conversely, SARS-CoV-2 nsp1-K164A had no significant effects on the host cell cycle. We also ruled out the possibility that SARS-CoV-2 nsp1 regulated the cell cycle by affecting the toxicity of host cells (Fig. 6c), confirming that the effect on the host cell cycle depends on the ability of SARS-CoV-2 nsp1 to regulate host proteins. These data show that nsp1 is the protein responsible for SARS-CoV-2-induced cell-cycle manipulation.

## Discussion

Many of the emerging pathogens that infect humans originate in animals, and coronaviruses (CoVs) are among such pathogens [27–29]. From the outbreak of SARS-CoV in 2002–2003 to the MERS-CoV in 2012 and then to the current SARS-CoV-2 pandemic, these infections cause great harm to the economy and public health [30–32]. As of 24 June 2020, newly emerged SARS-CoV-2 had spread to 216 countries, with more than 9.0 million confirmed cases and 469 000 deaths. Despite the large number of related studies, the focus has largely involved basic sequence analysis and drug screening [33–35], whereas the mechanisms of SARS-CoV-2 proteins are unclear.

Host translational suppression is an effective virus defence strategy that can inhibit the innate immune response of the host to finish viral protein proliferation [36, 37]. In acute viral infection, host cells shut down the translation system to cope with the infection stress, which is considered as a comprehensive stress response. Viruses have evolved a variety of effective strategies to support viral replication, such as utilizing either phosphorylation of eukaryotic initiation factor (eIF), the degradation cellular mRNAs, etc. African swine fever virus (ASFV) tended to enhance cap-dependent translation via the regulation of ATK-mTOR-4EBP1 pathway [38]. Us3 were encoded during Herpes Simplex Virus - 1 (HSV-1) infection to phosphorylate Tuberous Sclerosis Complex 2 (TSC2), which could active mTORC1 to promote eIF4E release [39]. Hepatitis C Virus (HCV) boosted the viral protein cap-dependent translation efficiency by expressing NS5A to active mTORC1 and eIF4E [40]. Influenza A viruses expressing shut-off-active NS1 with a reduced amount of PA-X expression most efficiently suppresses innate immune responses in host cells [41], and the herpes simplex virus (HSV) virion host shut-off (vhs) RNase suppresses host-protein synthesis and stimulates translation of viral mRNAs [42]. Poliocirus contain shut-off proteins like 2A protease that can stall the cap-dependent translation process by cleaving the eIF4G [43]. The phosphorylation of eIF2α during murine norovirus (MNV) infection is not responsible for the host shut-off [44]. CoV mRNAs are structurally equivalent to host mRNAs, which contain 5′-capped and 3′-polyadenylated. Due to the translation competition between cells and viruses, CoV must hijack the host translational machinery to produce its own protein. More and more studies focus on the translation control mechanism of CoV. Nsp1 promotes a variety of biological functions through inhibition of host translation [45, 46], which has a beneficial effect on the CoV. MERS-CoV nsp1 plays an important role in viral replication and possibly affects virus-induced diseases by promoting virus particle production in infected hosts [47]. In addition, PEDV nsp1 inhibits the type I IFN response by interfering with IRF3 and interrupts enhancer assembly by degrading the cap-binding protein (CBP) [48, 49]. Moreover, MHV nsp1 efficiently targets the type I IFN system and is important for protein processing, viral RNA synthesis and viral replication [21, 50]. SARS-CoV nsp1 has been studied with regard to the regulation of host-protein translation: it can inhibit expression of constitutive promoters and block translation of both host and virus mRNAs by binding to the 40S ribosomal subunit [24]. In addition, the complex consisting of SARS-CoV nsp1 and 40S ribosomal subunits might promote degradation of the 5′ cap region of mRNA [25]. Although the nsp1 amino acid sequence homology between SARS-CoV-2 and SARS-CoV is as high as 84 %, the detailed function of SARS-CoV-2 nsp1 remains unexplored.

In previous studies, CoVs nsp1 was demonstrated as an important virulence factor. Mutation of the motif at amino acids 91–95, the key functional region of TGEV nsp1, does not affect replication of the virus in cells but does reduce its virulence in susceptible animals [20]. Mutation of the functional region of nsp1 in MHV and SARS-CoV renders the virus less virulent, which is helpful for the development of attenuated vaccines [21, 22]. In this study, we first confirmed that SARS-CoV-2 nsp1 had the ability to inhibit host gene expression, similar to SARS-CoV nsp1 but with a stronger inhibitory effect. Then, we found that the SARS-CoV-2 nsp1 had little effect on the degradation of Rluc mRNA, which was consistent with the recently report [26]. Taken together, we speculated that SARS-CoV-2 nsp1 allowed the inhibition of host-protein synthesis as a consequence of its translational control activities. Furthermore, we determined that the key residue of SARS-CoV-2 nsp1 for host gene expression inhibition was K164, laying a solid foundation for the design of SARS-CoV-2 attenuated vaccines based on nsp1 modifications. By exploring how SARS-CoV-2 nsp1 controls host cells, we confirm that although SARS-CoV-2 nsp1 has no toxic effect, it promotes host-cell retention in G0/G1 phase. Many viruses, such as Coxsackievirus A6 and Murine norovirus-1, can induce cell-cycle arrest in G0/G1 phase [51, 52], providing a favourable environment for viral production. Furthermore, the viral non-structural protein 3D of Enterovirus D68 (EV-D68) are demonstrated to be responsible for the G0/G1 phase arrest prevents, which can promote viral production [53]. These results indicate that SARS-CoV-2 nsp1 manipulates the host cell cycle to arrest cells in the G0/G1 phase, which may be an important factor in promoting viral production.

In conclusion, we demonstrate that SARS-CoV-2 nsp1 inhibits host-gene expression by binding to the 40S ribosomal subunit via K164. The observed similarities reflect the homology between SARS-CoV-2 and SARS-CoV, but SARS-CoV-2 nsp1 also has its own specificity. For example, the inhibitory effect on host-protein synthesis is obviously stronger than that of SARS-CoV nsp1. Overall, our experimental data greatly contribute to our understanding of the mechanism of SARS-CoV-2 proteins. Regulation by SARS-CoV-2 nsp1 facilitates the development of a favourable environment in host cells, which may be of great help in designing SARS-CoV-2 attenuated vaccines.

## Methods

### Cell line and transfection

Human embryo kidney 293T (HEK-293T) cells were purchased from American Type Culture Collection (ATCC) and cultured in high-glucose Dulbecco’s modified Eagle’s medium (DMEM) supplemented with 100 U ml^−1^ penicillin, 10 µg ml^−1^ streptomycin and 10 % FBS (Invitrogen) at 37 °C with 5 % CO_2_ in a humidified incubator. Plasmids were transfected into HEK-293T cells by using Lipofectamine 2000 (Invitrogen) as described previously [51]. Briefly, the cells were washed with PBS three times before transfection. At 6 h post-transfection, the medium was replaced with fresh DMEM containing 3 % FBS.

### Antibodies

The following antibodies were used in this study: mouse anti-hemagglutinin (HA) monoclonal antibody (Proteintech); horseradish peroxidase (HRP)-conjugated goat anti-mouse IgG (Proteintech); mouse anti-puromycin monoclonal antibody (Millipore); goat anti-mouse IgG polyclonal antibody conjugated with Alexa Fluor 488 (Thermo Fisher Scientific); mouse anti-β-actin monoclonal antibody (Proteintech); rabbit anti-RPS6 polyclonal antibody (ABclonal); rabbit anti-RPS18 polyclonal antibody (ABclonal); rabbit anti-RPS2 polyclonal antibody (ABclonal); rabbit anti-RPS3 polyclonal antibody (ABclonal); rabbit anti-RPS8 polyclonal antibody (ABclonal); rabbit anti-RPS9 polyclonal antibody (ABclonal); rabbit anti-RPL30 polyclonal antibody (ABclonal).

### Plasmid construction

A mammalian expression vector, PCAGGS, was used to construct plasmids expressing various viral proteins and derived mutants. Constructs PCAGGS-HA-SARS-CoV-2 nsp1 and PCAGGS-HA-SARS-CoV nsp1 were generated by inserting DNA fragments encoding the SARS-CoV-2 nsp1 or SARS-CoV nsp1 protein with an N-terminal HA tag sequence. DNA fragments containing nsp1 mutations were cloned into PCAGGS-HA-SARS-CoV-2 nsp1 and PCAGGS-HA-SARS-CoV nsp1 using EcoRI and XhoI restriction sites.

### Indirect immunofluorescence assay (IFA)

IFA was performed to detect the localization of SARS-CoV-2 nsp1, SARS-CoV-2 nsp1-K164A and SARS-CoV nsp1 in HEK-293T cells. To examine expression of α-CoV nsp1 in HEK-293T cells, cells (2×10^5^/well) seeded in 24-well plates (Nest) were transfected with the corresponding plasmids for 24 h. Cells harbouring the empty PCAGGS vector were used as a control. Briefly, cells were fixed with 4 % paraformaldehyde for 10 min and permeabilized with precooled Triton X-100 for 10 min at room temperature. After three washes, the cells were blocked with 5 % bovine serum albumin in PBS for 1 h at 37 °C and incubated with anti-HA antibodies (1 : 1000; Proteintech) for 1 h at 37 °C. After four washes with PBST, a goat anti-mouse IgG polyclonal antibody conjugated with Alexa Fluor 488 (1 : 2000; Thermo Fisher Scientific) was added for 40 min at 37 °C. Nuclei were stained with 4′,6-diamidino-2-phenylindole (DAPI) (Sigma) for 10 min at room temperature. The samples were mounted in PBS and imaged using an LSM 510 Meta confocal laser-scanning microscope (Carl Zeiss, Zena, Germany).

### RNA extraction and quantitative real-time PCR (qPCR)

To determine the effect of SAR-CoV-2 nsp1 on Rluc mRNA, HEK-293T cells in 24-well plates were transfected with 0.5 µg nsp1 expression plasmid and 0.1 ug pRL-SV40. After 24 h, total RNA was extracted from transfected cells with TRIzol reagent (Invitrogen). RNA was then reverse transcribed into cDNA by avian myeloblastosis virus reverse transcriptase (TaKaRa, Japan). qPCR experiments were performed in triplicate. mRNA expression levels were normalized to an endogenous 18S rRNA. All qPCR primers used in this study are listed in Table 1.

**Table 1.** Primers used for qPCR

Primer*	Sequence (5′ to 3′)
Rluc-F	GCCAGTAGCGCGGTGTATTA
Rluc-R	AAATGCCAAACAAGCACCCC
18 S-F	AACCTGGTTGATCCTGCCAGT
18 S-R	GATCCTTCTGCAGGTTCACCTAC

*F, forward; R, reverse.

### CO-Immunoprecipitation (Co-IP)

HEK-293T cells that had been cultured in 60 mm dishes were cotransfected with the indicated expression plasmids expressing an HA tag. The cells were lysed in IP buffer (50 mM Tris-HCl [pH 7.4], 150 mM NaCl, 1 % NP-40, 10 % glycerin, 0.1 % SDS and 2 mM Na2EDTA) with rotation at 4 °C for 30 min. The cell lysates were then clarified by centrifugation at 13 000 r.p.m. at 4 °C for 20 min, and the supernatants were mixed with HA beads (Millipore) with rotation for 3 h at 4 °C. Thereafter, bead-bound immune complexes were collected using a DynaMag-2 magnet (Thermo Fisher Scientific) and washed five times with chilled PBS containing 0.1 % Tween 20. Finally, the bead-bound immune complexes were eluted by boiling in SDS-PAGE loading buffer at 100 °C for 10 min. The samples were analysed by Western blotting.

### Cell-viability assay

HEK-293T cells cultured in white 96-well plates (Corning, Tewksbury, MA, USA) were transfected with plasmids (0.2 µg) using Lipofectamine 2000. At 36 h post-transfection, cell viability was evaluated using the CellTiter-Glo luminescent cell viability assay reagent (Promega, Madison, WI, USA) following the manufacturer’s protocol. Briefly, an equal volume (100 µl) of CellTiter-Glo reagent was added, and the reaction mixture was incubated for 2 min on an orbital shaker and then for an additional 10 min at room temperature. The luminescence of each well was measured using a multimode plate reader (FLUOstar Omega, German). The percentage of cell viability was calculated as follows: percentage of cell viability=100× (luminescence of the experimental group/luminescence of the control group).

### Luciferase and ribopuromycylation assays

To measure the effect of nsp1 on the amount of Rluc production by HEK-293T cells, a luciferase assay was performed as described previously (48). Briefly, luciferase activity was determined by using a luciferase assay system (Promega, Madison, WI, USA) and a multimode reader (FLUOstar Omega, Germany). The ribopuromycylation assay was performed as described previously. Briefly, cultures of HEK-293T cells were transfected with a plasmid expressing wild-type SARS-CoV-2 or SARS-CoV nsp1 or mutants. At 36 h post-transfection, the cells were pulse-labelled with 3 µm puromycin and then incubated for an additional hour at 37 °C with 5 % CO_2_. After three washes with PBS, the cells were lysed via rotation at 4 °C for 30 min and subjected to Western-blot analysis.

### Flow cytometry

HEK-293T cells in the logarithmic growth phase were plated in a 12-well plate and transfected with related plasmids using Lipofectamine 2000. At 36 h post-transfection, the cells were washed twice with PBS and fixed with precooled 75 % ethanol using rotation at 4 °C overnight. The samples were examined using a BD FACSVerse (BD Biosciences, USA) following the instructions for Cell Cycle Detection Kit (Solarbio, 100 kit). The data were analysed using ModFit software.

### Statistical analysis

Statistical analysis was performed on the results of three independent experiments. Values are presented as the mean±SD. Statistical significance between different groups was calculated by GraphPad Prism Statistical Software. A two-tailed Student’s *t*-test was used. *** represents *P* <0.001.
